# CancerCostMod: a model of the healthcare expenditure, patient resource use, and patient co-payment costs for Australian cancer patients

**DOI:** 10.1186/s13561-018-0212-8

**Published:** 2018-10-31

**Authors:** Nicole Bates, Emily Callander, Daniel Lindsay, Kerrianne Watt

**Affiliations:** 1 College of Public Health, Medical and Veterinary Sciences (CPHMVS), James Cook University, Townsville, Australia; 2 Australian Institute of Tropical Health and Medicine (AITHM), James Cook University, Townsville, QLD Australia

**Keywords:** CancerCostMod, Cancer, Economic burden, Cost, Healthcare system, Patient co-payment

## Abstract

Although cancer survival in general has improved in Australia over the past 30 years, Indigenous Australians, socioeconomically disadvantaged persons, and people living in remote areas still experience poorer health outcomes. This paper aims to describe the development of CancerCostMod, and to present the healthcare expenditure and patient co-payments for the first 12-months post-diagnosis. The base population is a census of all cancer diagnoses (excluding non-melanoma skin cancer) in Queensland, Australia between 1 July 2011 and 30 June 2012 (*N* = 25,553). Each individual record was linked to their Queensland Health Admitted Patient Data Collection, Emergency Department Information System, Medicare Benefits Schedule, and Pharmaceutical Benefits Scheme records from 1 July 2011 to 30 June 2015. Indigenous status was recorded for 87% of participants in our base population. Multiple imputation was used to assign Indigenous status to records where Indigenous status was missing. This base population was then weighted, using a programmed SAS macro (GREGWT) to be representative of the Australian population. We adopted a national healthcare perspective to estimate the cost of cancer for hospital episodes, ED presentations, primary healthcare, and prescription pharmaceuticals. We also adopted an individual perspective, to estimate the primary healthcare and prescription pharmaceutical patient co-payments. Once weighted, our sample represents approximately 123,900 Australians (1.7% Indigenous Australians). The total healthcare system cost of all cancers during the first 12-months post diagnosis was $4.3 billion, and patient co-payments costs were $127 million. After adjusting for sex, age at diagnosis, Indigenous status, rurality, socioeconomic status, and broad cancer type, significant differences in costs were observed for population groups of interest within the first year post-diagnosis. This paper provides a more recent national estimate of the cost of cancer, and addresses current research gaps by highlighting the distribution of healthcare and individual costs by Indigenous status, rurality, and socioeconomic status.

## Introduction

In Australia, the overall cancer mortality rate decreased by 23% between 1982 and 2017 [1]. However, three population groups experience poorer cancer outcomes compared to the general Australian population: Australian Aboriginal and/or Torres Strait Islander people (hereafter respectfully referred to as Indigenous people), socioeconomically disadvantaged persons, and people living in remote areas [1]. To add to the complexity of this issue, these population groups often overlap. Indigenous people more often live in remote and very remote areas [2], and a greater proportion of people living in rural and remote areas experience socioeconomic disadvantage [3]. A recent study found that the socioeconomic disparities in cancer survival appear to have worsened over the past 30 years. The gap remained after accounting for diagnosis stage, and cancer site [4].

A number of factors have been identified in contributing to these survival inequalities, including but not limited to differences in risk factors [1, 5], being diagnosed at a later stage [5, 6], differences in access to oncology services [7, 8], a greater number of comorbidities [6], and differences in treatment [6, 9–11]. High patient out-of-pocket expenditure (OOPE) may also impact a person’s decision to access care, with one study finding 21% of people with cancer skipping healthcare due to the cost [12], and another study found that 11% of patients found that prescriptions relating to their oncology treatment caused financial burden [13]. A recent study reported that the median total cost to the patient for provider fees during the first 2 years was over $20,000 for people diagnosed with lung or breast cancer [14]. Studies using self-reported costs, have found that OOPE is higher for those who have to travel to receive treatment, due to travel and accommodation costs [15, 16].

The healthcare system expenditure for cancer is also high [17–19]. A commissioned report estimated that the lifetime costs of cancer for a patient diagnosed in New South Wales (NSW), Australia, in 2005 was $3.9 billion [17]. These costs are expected to rise with new technologies, new pharmacotherapies, and population changes [20]. This predicted increase in costs has already been seen with the annual Pharmaceutical Benefits Scheme (PBS) expenditure on anticancer drugs increasing by approximately $400 million between 1999 and 2000 and 2010–11. This was an average increase of 19% compared to 9% for all other drugs combined [21]. The Australian Institute of Health and Welfare (AIHW) reported that cancer cost approximately $4.5 billion in 2008–09 (excluding screening). The majority of these costs were due to hospital admitted patient services (79%), followed by prescription pharmaceuticals (12%), and out-of-hospital costs, such as general practitioner, specialists, pathology, and imaging (9%) [18]. However, these reports are now a decade old, and do not look at the distribution of costs by population group. To the author’s best knowledge, only one report has compared the cost of hospital expenditure by Indigenous status. The AIHW and Cancer Australia reported that in 2010–11, the per person hospital expenditure for all cancers combined for Indigenous Australians was half of that for non-Indigenous Australians [19].

CancerCostMod is Australia’s first model of health service use, healthcare expenditure, and patient co-payments for people diagnosed with cancer in Australia. This model has several objectives:

1. To quantify the current health system use, and healthcare expenditure for people with cancer, and to determine any inequalities by Indigenous status, socioeconomic status, and remoteness;
2. To quantify the patient co-payment for people with cancer, and to determine any inequalities by Indigenous status, socioeconomic status, and remoteness;
3. To estimate the costs to government and individuals of “optimal service use” - if all patients received the maximum access of care.

This paper aims to 1) describe the development of our model, CancerCostMod; 2) to describe the total costs of cancer in Australia during the first 12-months post-diagnosis; and 3) to describe the distribution of the cost of cancer in Australia during the first 12-months post-diagnosis by population group.

## Methods

### Base population – Linked administrative data

The protocol describing the data linkages that were undertaken to create this dataset has been described previously [22]. Briefly, the base population for this model was a census of all patients diagnosed with cancer in Queensland between 1 July 2011 and 30 June 2012, as recorded by the Queensland Cancer Registry (QCR) (*N* = 25,553 patients). Each individual’s QCR record was linked to their Queensland Health Admitted Patient Data Collection (QHAPDC) records (243,034 separations for 21,944 patients), and Queensland Health Emergency Department Information Systems (EDIS) (46,455 presentations from 12,825 patients) from 1 July 2011 to 30 June 2015. This linkage was conducted by the Queensland Health Statistical Services Branch using deterministic and probabilistic methods. Each participant’s record was then linked to their individual Medicare Benefits Schedule (MBS) (6,058,380 services) and Pharmaceutical Benefits Scheme (PBS) (2,619,712 prescriptions) records from 1 July 2011 to 30 June 2015 by the Data Linkage Unit at the AIHW using probabilistic linkage. Overall, 99.4% of cohort members were matched to the Medicare enrolments register.

The QCR dataset contained date of diagnosis, International Classification of Diseases for Oncology (ICD-O) morphological code, ICD-O topographical code, differentiation code, behaviour status, as well as date and cause of death if applicable. The stage at diagnosis is not routinely collected by the QCR. Other variables included sociodemographic information, such as date of birth, sex, Indigenous status, and postcode at diagnosis.

The QHAPDC records contained information on all separations from a private or public hospital in Queensland. Facility number, patient residential postcode, admission and separation date, length of stay, hospital insurance status, and the Australian Refined Diagnostic-Related Group (AR-DRG) was recorded for each separation. The AR-DRG is a classification system, where episodes of care are coded using the ICD-10-Australian Modification (ICD-10-AM) and Australian Classification of Health Interventions, which is used to code procedures and interventions [23]. The EDIS dataset contained all emergency department (ED) presentations, including information such as date of presentation, facility number, patient postcode at time of presentation, triage category (Australasian Triage Scale for treatment prioritisation), discharge destination, and ICD-10-AM code.

MBS data include individual patient identifier, patient postcode, date of service, provider postcode, MBS item code, fee charged, benefit paid (by MBS), patient co-payment costs, and hospital flag. MBS claims data exclude Department of Veteran’s Affairs beneficiaries. PBS data includes individual patient identifier, patient postcode, date of supply, PBS item, patient category (concession or general), patient co-payment costs, benefit amount, pharmacy postcode. PBS data excludes private prescriptions, over-the-counter medications, under co-payment prescriptions, Repatriation Pharmaceutical Benefits Scheme prescriptions, and any medications dispensed under special arrangements.

### Development of CancerCostMod

#### Cancer classification

Using the ICD-O 3rd Edition [24] and the Cancer Council Queensland methods website [25], the type of cancer was grouped into 18 broad cancer categories: head and neck; digestive organs; colorectal cancer; female genital organs; breast cancer; prostate cancer; male genital organs excluding prostate; urinary tract; eye, brain and other parts of the central nervous system (CNS); mesothelioma, Kaposi sarcoma and soft tissue; thyroid and other endocrine organs; other thoracic and respiratory organs; bone; tracheal, bronchus and lung cancer; other skin; melanoma; blood and lymphatic system; other or ill-defined cancers.

#### Socioeconomic status and rurality

The patient’s postcode at diagnosis was mapped to the Australian Bureau of Statistics (ABS) Index of Relative Socio-Economic Disadvantage (IRSD), and Australian Statistical Geography Standard (ASGS) [26]. IRSD is a summary of the economic and social conditions of an area, and is a measure of relative disadvantage only, where decile 1 was the most disadvantaged, and decile 10 was the least disadvantaged. ASGS has five categories ‘metropolitan’, ‘inner regional’ ‘outer regional’, and ‘remote’ and ‘very remote’. There were 151 records with ‘unknown or not stated’ postcodes at diagnosis, which could not be mapped to IRSD or rurality. We have categorised these records as ‘unknown or not recorded’ for IRSD and rurality. For our analyses, we collapsed rurality into three categories (‘metropolitan’, ‘regional’, and ‘remote’) and IRSD into quintiles (Q1: most disadvantaged and Q5: least disadvantaged).

#### Indigenous status

A common data limitation in Australia is that Indigenous status may be incompletely recorded in health and vital registration data collections [27]. The Australian Cancer Database considers five (of eight) jurisdictions to have sufficient completeness for reporting purposes, NSW, Victoria, Queensland, Western Australia and the Northern Territory [1]. A 2011–12 audit found that Indigenous status in Queensland Health hospital admission records had 87% weighted completeness (CI 84–91%) [27].

Indigenous status was recorded for 87% of participants in our original cohort from the QCR dataset. We used a number of methods to assign Indigenous identification to the 13% of records for which there was missing data or ‘unknown’ Indigenous status recorded (*n* = 3316). Initially, records with missing or unknown Indigenous identification on the QCR dataset were assigned to be ‘Indigenous’ if they resided in a local government area (LGA) that had greater than or equal to 75% of the population identified as Indigenous, as reported by the ABS’ estimated resident Indigenous Australian and non-Indigenous Australian populations in each Queensland LGA for 2011 [28]. Seventy-five percent was chosen as a conservative cut-off, reflecting the definition by the ABS of a ‘Discrete Indigenous Community’ as one that has greater than half of the population identifying as Aboriginal and/or Torres Strait Islander [29].

We then used multiple imputation (MI) to impute the remaining missing values for Indigenous status (*n* = 3297). We used the MI procedure in SAS 9.4 (SAS Institute Inc., Cary, NC, USA), which has three distinct phases [30]. We used logistic regression to develop the imputation model for multiple imputation, as the variable of interest was dichotomous (Indigenous or not-Indigenous). Covariates used were similar to those used by Morell et al. [31], and included sex, 5-year age group, 12-month survival, country of birth (Australia, not Australia, or not stated/unknown), broad cancer group (18 categories), rurality (5 categories), and IRSD deciles. We used PROC MI with a monotone logistic statement, with 20 imputations, followed by PROC LOGISTIC, and finally PROC MIANALYZE to produce inferential results.

#### Weighting to the Australian population

In order to provide results that are representative to the Australian population, we used the programmed SAS macro GREGWT to weight our dataset to the Australian population. GREGWT is a generalised regression reweighting algorithm which was developed by the ABS and is commonly used to weight their household surveys against known benchmarks [32]. The mathematical techniques underlying GREGWT have been described elsewhere [33, 34]. In the current study, data were benchmarked against the 2012 Australian Cancer Database [35]. This database provides Australia-wide cancer incidence rates stratified by cancer type, age group and gender. Cancer incidence rates for 2012 were extracted from this database and used as the benchmark for the calculation of weights in the current study.

### Defining costs

We developed CancerCostMod to allow analyses of costs from different perspectives. We adopted a national healthcare perspective, to estimate the direct cost of cancer to the healthcare system for hospital, primary healthcare and prescription pharmaceuticals. We also adopted an individual perspective, to estimate the patient co-payments for primary healthcare and prescription pharmaceuticals.

All costs were calculated monthly for each individual from the date of diagnosis (*t* = 0) for 36 months. If an individual had no health services for the month, the cost was recorded as $0. All costs are reported in Australian dollars (AUD), and were adjusted to the 2016–17 financial year using the Reserve Bank of Australia inflation calculator [36].

#### Hospital costs

Within Australia, public hospitals are run by the State and Territory governments, and are jointly funded by the state and national governments through either activity-based funding (ABF) or block funding [37]. The ABF model pays hospitals for the number and mix of services provided, and accounts for patients that may be more complicated. The Independent Hospital Pricing Authority (IHPA) was established as part of the National Reform Act 2011, and is responsible for the implementation of the ABF for public hospitals. The IHPA produces an annual National Hospital Cost Data Collection (NHCDC), which includes the voluntary submission of cost data for each AR-DRG from public and private hospitals. This information is then used to develop the National Efficient Price, which determines the federal contribution to the ABF system, and the National Efficient Cost, which determines the federal contribution to block funding. Each State and Territory is then responsible for distributing the federal and state funding to their public hospitals. The annual NHCDC reports are available online [38–41]. Private hospitals are owned and operated by private institutions but must still comply with national standards. Private hospitals receive funding from patient charges, private insurance, and Medicare rebates [42].

The cost attributed to each AR-DRG for public hospital separations was assigned using the actual cost as reported by the NHCDC Report (available online) [38–41] for the relevant year. To reflect possible variations in the costs of delivering healthcare to some individuals, we included the adjustment for certain patient demographics produced by the IHPA (pediatric, patient remoteness and/or Indigenous person, and private patient service and accommodation) [43–45]. As there were no adjustments published for the 2011–12 financial year, we used the adjustment for the 2012–13 period. The cost attributed to each AR-DRG for private hospital separations was assigned for the relevant year using the average charge per separation reported by the Private Hospital Data Bureau Annual Reports (available online) [46].

In Australia, some patients may receive treatment as a non-admitted (out-) patient at a hospital location, but are not formally ‘admitted’, and are therefore not captured in the QHAPDC dataset. In order to capture these non-admitted hospital services, we included MBS items which related to services or procedures performed in a hospital setting. This method has been used in previous Australian studies [47, 48]. We identified these MBS items by conducting a keyword search of the MBS item descriptions to identify hospital items: ‘hospital’ (excluding codes which specified it was in a place ‘other than a hospital’), ‘theatre’, ‘emergency department’, and ‘prior to discharge’. Item codes relating to chemotherapy and radiotherapy were also identified through consultation with staff from the Townsville Hospital and Health Service, Townsville Cancer Centre. The rebate paid by Medicare for MBS codes for these ‘hospital’ items were included in the hospital costs. Hereafter, admitted and non-admitted hospital episodes will be referred to as ‘hospital episodes’.

The ED classification system, Urgency Disposition Groups, and Urgency Related Group (URG), was originally developed in Western Australia [49] to group patient presentations into categories. The IHPA implemented this system nationally in 2012, and it has since undergone several revisions commissioned by the IHPA [50]. Each ED presentation was coded to a URG using the triage category, discharge destination and the primary reason for attending the ED (ICD-10-AM). The cost attributed to each URG for each ED presentations was assigned using the average cost per presentation as reported by the NHCDC Report (available online) [38–41] for the relevant year.

#### MBS and PBS costs

Briefly, Australia’s universal healthcare system (Medicare), provides free and subsidized medical services under MBS and PBS. For services covered by MBS, if there is a gap between the rebate paid by Medicare and the amount charged by the service provider, the individual will incur an out-of-pocket co-payment. In some cases, the service provider may ‘bulk-bill’ a patient, or charge the amount equal to the Medicare rebate, resulting in no individual co-payment. For prescription pharmaceuticals, the individual will be charged up to the set patient co-payment for concession (low income card holders) and general patients. Australia has several safety net arrangements for individuals and family groups with high OOPE (two Medicare Safety Nets, and one PBS Safety Net). Once an individual or family group reaches a given amount on co-payments for a calendar year, they will receive a higher government subsidy, resulting in reduced patient co-payments [51]. Furthermore, Indigenous Australians living with, or at risk of chronic disease may also be eligible to receive prescription pharmaceuticals at reduced cost through the Closing the Gap (CTG) PBS Co-payment Programme [52]. However, hospital prescriptions are excluded from this programme [52].

The MBS and PBS data includes date of service/dispensing, MBS/PBS item number, patient postcode, provider/pharmacy postcode, and total fee charged, rebate paid, and patient co-payment.

### Statistical analysis

Initially, descriptive analysis was undertaken to describe the social and demographic characteristics of the sample, and weighted sample. Then the total cost for the first 12-months post-diagnosis for the five types of healthcare expenditure: hospital episodes, ED presentations, MBS items, PBS items, and patient co-payments are presented for the broad type of cancer, and the population groups of interest. We also present the average total healthcare cost per person (to the Australian healthcare system), and the average patient co-payment per person during the first 12-months following a diagnosis. We chose to include the standard deviation, as in many cases, the standard deviation was greater than the mean, indicating a wide dispersion of the costs.

Finally, the total cost of cancer for the first 12-months following diagnosis for each of the five types of healthcare were modelled with a generalized linear model using a gamma distribution, with a log link function. This included the number of months the patient survived as an offset to the model. The analysis was limited to include adults (≥ 18 years at diagnosis), and only to those who had costs for the healthcare type used in the model. Independent variables included in the analysis were sex, age at diagnosis, Indigenous status (reference group = non-Indigenous Australians), rurality (three categories; reference group = metropolitan), IRSD quintiles (reference group = Q1 (most disadvantaged)) and broad cancer type (18 categories; reference group = tracheal, bronchus and lung cancer).

All analyses were undertaken using SAS V9.4 (SAS Institute Inc., Cary, NC, USA). Weighted estimates are presented, unless otherwise stated.

Human Research Ethics approval was obtained from the Townsville Health and Hospital Service Human Research Ethics Committee (HREC) (HREC/16/QTHS/11), AIHW (EO2017/1/343) and James Cook University HREC (H6678). Permission to waive consent was approved from Queensland Health under the Public Health Act 2005. No identifiable information was provided to the authors.

## Results

### CancerCostMod cancer incidence

In total, 25,553 individuals were diagnosed with a new cancer in Queensland between July 2011 and June 2012. Once weighted, this represents 123,900 Australians. Table 1 reports the demographic characteristics of our model for all cancers, and for the five most commonly diagnosed cancers in Australia. Our original QCR dataset had complete Indigenous status for 87% of our records, which matches an audit of Queensland Health hospital admission records in 2011–12 [27]. After imputation, we estimated that 2129 of our model were Indigenous Australians.

**Table 1:** Demographic characteristics of CancerCostMod for all cancers combined, and most commonly diagnosed cancers^a^

	All cancers	Breast cancer	Prostate cancer	Colorectal cancer	Tracheal, bronchus and lung cancer	Melanoma of the skin
Actual n	25,600	3100	4200	2900	2100	3300
Weighted N (%)	123,900	15,400 (12.5)	20,700 (16.7)	14,800 (11.9)	11,100 (9.0)	12,300 (9.9)
Died within 12-months post-diagnosis (%)	25,100 (20.2)	700 (4.2)	1000 (4.6)	2400 (16.3)	6400 (57.7)	400 (3.1)
Age group						
≤ 17 (%)	1000 (0.8)	–	–	–	–	–
18 to 44 (%)	9300 (7.5)	1800 (12.0)	100 (0.6)	700 (4.7)	200 (1.6)	1800 (14.6)
45 to 64 (%)	42,700 (34.5)	7600 (49.1)	7800 (37.7)	4100 (27.7)	2900 (26.3)	4500 (36.4)
≥ 65 (%)	70,900 (57.2)	6000 (38.9)	12,800 (61.8)	10,000 (27.6)	8000 (72.1)	6000 (48.7)
Sex						
Male (%)	69,300 (55.9)	100 (0.7)	20,700 (100)	8100 (54.9)	6500 (58.4)	7200 (58.4)
Female (%)	54,600 (44.1)	15,300 (99.3)	n/a	6700 (45.1)	4600 (41.6)	5100 (41.6)
Indigenous status						
Non-Indigenous Australian (%)	121,800 (98.3)	15,200 (98.4)	20,500 (99.0)	14,600 (99.1)	10,800 (97.3)	12,100 (98.8)
Indigenous Australian (%)	2100 (1.7)	300 (1.6)	200 (1.0)	100 (0.9)	300 (2.7)	100 (1.2)
Rurality^b^						
Metropolitan (%)	58,500 (47.5)	7800 (50.5)	9100 (44.1)	6700 (45.9)	5000 (45.2)	6100 (49.7)
Regional (%)	54,500 (44.2)	6400 (41.7)	9600 (46.5)	6600 (45.1)	5100 (45.8)	5300 (43.7)
Remote (%)	10,200 (8.3)	1200 (7.8)	1900 (9.4)	1300 (9.1)	1000 (9.0)	800 (6.7)
IRSD^c^ Quintiles^b^						
Q1 (Most disadvantaged) (%)	11,300 (9.2)	1100 (7.2)	2100 (10.2)	1400 (9.8)	1300 (11.6)	1000 (8.2)
Q2 (%)	5700 (4.7)	800 (5.0)	900 (4.3)	700 (4.6)	600 (5.3)	500 (4.3)
Q3 (%)	19,900 (16.2)	2500 (16.3)	3400 (16.5)	2500 (16.7)	1900 (17.5)	1800 (14.7)
Q4 (%)	56,000 (45.5)	6700 (43.7)	9300 (45.2)	6700 (45.7)	4900 (44.5)	5900 (47.9)
Q5 (Least disadvantaged) (%)	30,200 (24.5)	4300 (27.8)	4900 (23.8)	3400 (23.1)	2300 (21.1)	3000 (24.9)

^a^ Weighted results reported (except in the first row), and rounded to the nearest 100. Weighted values less than 100 are not reported

^b^ Excluding individuals with missing postcodes

^c^ Index of Relative Socio-Economic Disadvantage

Table 2 reports the age-standardised incidence rates for our weighted model, compared to the national age-standardised incidence rates for new cases diagnosed in 2012. The CancerCostMod age-standardised incidence rate for the five most commonly diagnosed cancers in Australia are similar to the national age-standardised incidence rate for new cases in 2012.

**Table 2:** Australian and CancerCostMod Cancer Incidence

Broad cancer group	National new cases, 2012	National incidence rate (a)	CancerCostMod cases	CancerCostMod incidence rate (a)
All cancers	121,693	482.85	123,915	492.75
Breast cancer (female only)	15,337	120.42	15,335	120.56
Prostate cancer (male only)	20,687	168.25	20,687	168.37
Colorectal cancer	14,793	58.46	14,774	58.54
Tracheal, bronchus and lung cancer	11,114	43.78	11,104	43.52
Melanoma of the skin	12,250	49.51	12,250	49.42

^(a)^ Incidence rates are standardised to the Australian population as at 30 June 2001 and are expressed per 100,000 population

### Cost of cancer during the first 12-months post-diagnosis by broad cancer type

The total initial cost associated with newly diagnosed cancer for the healthcare system was $4.3 billion, and total patient co-payment was $127.7 million. Hospital episodes accounted for 77% of the healthcare expenditure, followed by PBS (14%) and MBS 6%. Table 3 shows the total cost of cancer for the first 12-months following diagnosis for all cancers combined, and for each broad cancer type (excluding ‘bone’ and ‘other thoracic and respiratory organs’ cancers due to sample size). The most expensive cancers to the healthcare system were cancers of the blood and lymphatic system, followed by colorectal cancer and breast cancer. Colorectal cancer was the most expensive cancer in regards to hospital episodes, and cancers of the blood and lymphatic system had the highest MBS and PBS rebate costs. During the first 12-months, cancers of the eye, brain and other parts of the central nervous system (CNS) accounted for the highest average cost per person to the Australian healthcare system, followed by cancers of the blood and lymphatic system. The most expensive cancers in regards to patient co-payments were cancers of the blood and lymphatic system, followed by breast cancer and colorectal cancer. The average patient co-payment costs per person were highest for cancers of the blood and lymphatic system, followed by cancers of the eye, brain and other parts of the central nervous system (CNS).

**Table 3:** Total cost of cancer during the first 12-months post-diagnosis by cancer type^a^

		Cost To The Australian Healthcare System (AUD)						Patient co-payment^e^ (AUD)	
	N^b^	Hospital episodes^c^	ED presentations	MBS rebate^d^	PBS rebate	Total cost to the healthcare system	Average healthcare cost per person (SD)	Total	Average per person (SD)
All cancers combined	123,900	3,302,933,000	88,103,100	263,243,600	631,804,300	4,286,083,900	34,600 (41300)	127,673,700	1000 (2000)
Prostate cancer	20,700	279,788,000	6,416,200	36,828, 600	38,650,600	361,683,300	17,500 (17,300)	16,324,000	800 (1200)
Breast cancer	15,400	266,860,700	6,744,700	36,690, 600	170,956,200	481,252,200	31,200 (29,700)	21,040, 900	1400 (1500)
Colorectal cancer	14,800	581,675,500	11,849,400	30,563,000	71,878,900	695,966,800	47,100 (38,200)	16,979,500	1100 (1800)
Blood and lymphatic system	13,300	564,265,300	13,286,600	40,503,100	176,947,100	795,002000	59,800 (70,800)	24,655,400	1900 (4100)
Melanoma of the skin	12,300	53,047,300	1,575,700	23,174,800	12,506,700	90,304,500	7400 (12600)	4,945,100	400 (500)
Tracheal, bronchus and lung cancer	11,100	396,063,600	15,618,400	24,164,600	45,331,000	481,177,600	43,300 (34,700)	10,168,700	900 (1700)
Digestive organs	10,100	394,784,900	12,668,100	20,303,100	31,119,600	458,875,800	45,500 (36,800)	11,273,000	1100 (2000)
Cancers of the urinary tract	6200	181,697,200	5,022300	10,703,000	17,623,000	215,045,500	34,700 (31800)	4,816,000	800 (1100)
Gynaecological cancers	5200	138,156,900	3,121,600	10,475,000	16,774,700	168,528,200	32,300 (31500)	5,171,800	1000 (1600)
Head and neck	4000	118,588,400	2,478,200	10,264,700	9,019,700	140,351,100	34,900 (45,500)	2,374,400	600 (1200)
Other or ill-defined cancers	2700	67,701,500	2,316,900	4,295,300	6,051400	80,365,200	29,600 (29,700)	2,239,700	800 (1700)
Thyroid and other endocrine glands	2600	48,531,700	799,400	4,195,500	3,631,300	57,157,900	22,000 (34,400)	1,180,600	500 (600)
Eye, brain and CNS	1900	101,300,100	3,113,900	3,324,700	17,342,800	125,081500	64,700 (70,400)	2,830,000	1500 (2300)
Mesothelioma, Kaposi Sarcoma, and soft tissue	1600	62,604,600	1,882,200	3,245,200	9,187,200	76,919,200	47,100 (46100)	2,080,100	1300 (2200)
Male genital organs, exc prostate	900	17,921,400	678,900	2,190,200	2,267,400	23,057, 900	25,000 (30,400)	643,200	700 (1200)
Other skin cancer	800	11,920,400	283,300	1,749,400	1,456,800	15,409,900	18,600 (28100)	541,900	700 (1100)

^a^ Weighted results reported, and rounded to the nearest 100

^b^ Excluding broad cancer types with less than 500 individuals (‘bone’, and ‘other thoracic and respiratory organs’)

^c^ Admitted and non-admitted hospital episodes

^d^ MBS rebate, excluding items included as non-admitted hospital episodes

^e^ MBS and PBS patient co-payment

### Cost of cancer during the first 12-months post-diagnosis by population group

We then aimed to describe the cost of cancer for the first 12-months post-diagnosis by Indigenous status, rurality, and socioeconomic disadvantage, as shown in Table 4. The average total healthcare cost per person was greater for Indigenous people diagnosed with cancer in the first 12-months, compared to non-Indigenous people diagnosed with cancer. However, the average patient co-payment was greater for non-Indigenous people diagnosed with cancer, compared to Indigenous people diagnosed with cancer. Table 4 also shows that the average total healthcare cost per person was greater for people from the most disadvantaged quintile (Q1), and the lowest for people in the least disadvantaged quintile (Q5). Conversely, the average patient co-payment was greatest for people in the least disadvantaged quintile (Q5), and lowest for people in the most disadvantaged quintile (Q1).

**Table 4:** Total cost of cancer for the first 12-months post-diagnosis, by population groups^1^

		Cost To The Australian Healthcare System (AUD)						Patient co-payment^4^ (AUD)	
	n	Hospital episodes^2^	ED presentations	MBS rebate^3^	PBS rebate	Total cost to the healthcare system	Average healthcare cost per person (SD)	Total	Average per person (SD)
Weighted total	123,900	3,302,933,000	88,103,100	263,243,600	631,804,300	4,286,083,900	34,600 (41300)	127,673,700	1000 (2000)
Indigenous status									
Non-Indigenous Australian	121,800	3,233,868,900	85,643,100	259,009,200	623,618,600	4,202,139,700	34,500 (41100)	126,913,100	1000 (2000)
Indigenous Australian	2100	69,064100	2,459,900	4,234,500	8,185,700	83,944,200	40,100 (48100)	760,600	400 (800)
Rurality^5^									
Metropolitan	58,500	1,485,140,700	38,505,200	125,884,200	306,909,000	1,956,439,200	33,500 (38,200)	65,570,600	1100 (1900)
Regional	54,500	1,495,270,400	42,165,900	116,837,100	270,755,900	1,925,029300	35,300 (42300)	52,061400	1000 (1900)
Remote	10,200	311,295,100	6,805,500	19,718,200	52,844,900	390,663,700	38,200 (51900)	9,831,600	1000 (2500)
IRSD^6^ Quintiles^5^									
Q1 (Most disadvantaged)	11,300	349,061100	11,248,900	21,232,900	47,295,500	428,838,500	38,000 (43000)	9,226,300	800 (1900)
Q2	5700	160,726,000	3,180,500	11,653,000	28,801,500	204,361,000	35,700 (42300)	4900,200	900 (1800)
Q3	19,900	554,607,600	14,727,500	41,726,900	102,484,100	713,546,100	35,900 (46,800)	17,757,000	1100 (1800)
Q4	56,000	1,469,376,800	42,010,300	128,352,200	288,661,500	1,928,400,700	34,400 (40,400)	59,401,200	1100 (1800)
Q5 (Least disadvantaged)	30,200	757,934,600	16,309,300	59,474,600	163,267,300	996,985,900	33,000 (38,300)	36,179,100	1200 (2000)

^1^ Weighted results reported, and rounded to the nearest 100

^2^ Admitted and non-admitted hospital episodes

^3^ MBS rebate, excluding items included as non-admitted hospital episodes

^4^ MBS and PBS patient co-payment

^5^ Excluding individuals with missing postcodes

^6^ Index of Relative Socio-Economic Disadvantage

Finally, we conducted five generalized linear models to predict costs, limiting our analyses to adults only (≥ 18 years), adjusting for sex, Indigenous status, rurality, IRSD quintile, age at diagnosis, and broad cancer type (co-efficients not shown). Table 5 shows the abbreviated output for the five types of cost: hospital episodes, ED presentations, MBS rebates (excluding hospital items), PBS rebates, and patient co-payments. Indigenous Australians had significantly higher costs for hospital episodes (22% higher) and ED presentations (23% higher), but significantly lower costs for MBS rebates (8% lower), PBS rebates (18% lower), and patient co-payments (61% lower) compared to non-Indigenous Australians. The costs for hospital episodes had significantly higher costs in the first 12-months post-diagnosis with increasing remoteness, 6% for people living in inner and outer regional areas, and 15% higher for people living in remote and very remote areas compared to those living in metropolitan areas. People living in remote and very remote areas had 10% lower costs for MBS rebates and 10% higher PBS rebate costs. Compared to those living in the most disadvantaged areas (IRSD Q1), those in quintiles 3–4 had decreasing costs associated with hospital episodes. There were no differences in the costs of ED presentations for IRSD quintiles. The costs incurred for MBS and PBS rebates and also patient co-payments increased as the IRSD quintile increased (moved towards the least disadvantaged).

**Table 5:** Five generalized linear models of the cost of cancer for the first 12-months^1^

	Hospital episodes^2^		ED presentations		MBS rebate^3^		PBS rebates		Patient co-payment^4^	
	Ratio	Estimate (SE)	Ratio	Estimate (SE)	Ratio	Estimate (SE)	Ratio	Estimate (SE)	Ratio	Estimate (SE)
Intercept	–	8.4819 (0.0526) ***	–	5.4363 (0.0728) ***	–	5.7499 (0.0382) ***	–	6.8536 (0.0721) ***	–	4.7398 (0.0593) ***
Age	1.01	0.0073 (0.0005) ***	1.01	0.0103 (0.0008) ***	1.00	−0.0021 (0.0004) ***	0.99	−0.0109 (0.0008) ***	1.00	0.0033 (0.0006) ***
Female	0.89	−0.1177 (0.0186) ***	0.95	−0.0537 (0.0262) *	0.95	−0.0551 (0.0129) ***	0.85	−0.1597 (0.0234) ***	0.98	−0.0214 (0.0192)
Indigenous Australians	1.22	0.1978 (0.0605) **	1.23	0.2095 (0.0752) **	0.92	−0.0888 (0.0402) *	0.82	−0.1928 (0.0735) **	0.39	−0.9315 (0.0619) ***
Inner and outer regional area	1.06	0.0557 (0.0182) **	1.02	0.0196 (0.0275)	0.99	−0.0138 (0.0126)	1.04	0.0366 (0.0231)	0.94	−0.0571 (0.0189) **
Remote and very remote area	1.15	0.1401 (0.0296) ***	1.03	0.0322 (0.0454)	0.90	−0.1092 (0.0207) ***	1.10	0.0912 (0.0381) *	0.98	−0.0219 (0.0311)
IRSD^5^ Q2	1.01	0.0091 (0.0415)	1.00	0.0004 (0.0626)	1.07	0.0675 (0.0289) *	1.05	0.0474 (0.0519)	1.00	−0.0026 (0.0432)
IRSD^5^ Q3	0.94	−0.0619 (0.0309) *	1.01	0.0097 (0.0420)	1.08	0.0805 (0.0216) ***	1.11	0.1053 (0.0389) **	1.00	−0.0020 (0.0324)
IRSD^5^ Q4	0.90	−0.1042 (0.0275) ***	0.98	−0.0215 (0.0368)	1.18	0.1642 (0.0193) ***	1.17	0.1582 (0.0348) ***	1.23	0.2033 (0.0289) ***
IRSD^5^ Q5 (least disadvantaged)	0.88	−0.1297 (0.0323) ***	0.94	−0.0603 (0.0467)	1.00	0.0016 (0.0226)	1.19	0.1703 (0.0411) ***	1.32	0.2746 (0.0338) ***

* p-value < 0.05

** *p*-value < 0.01

*** p-value < 0.001

^1^ Adjusted for age at diagnosis, sex, Indigenous status, rurality, IRSD quintiles, and broad cancer type

^2^ Admitted and non-admitted hospital episodes

^3^ MBS rebate, excluding items included as non-admitted hospital episodes

^4^ MBS and PBS co-payment is the cost to the patient

^5^ Index of Relative Socio-Economic Disadvantage

## Discussion

The use of administrative health data is growing in Australia and is supported by a number of Australian Government agencies [53]. There are many advantages of using administrative data for research, including being non-intrusive to the target population, no (or low) cost for data collection (but there may be a cost-recovery charge), and the ability to capture a large population. Administrative data has advantages over sample data, which relies on self-reported information from patients, and also excludes patients who have passed away. There are a growing number of international studies which have used linked administrative data to describe the patterns and cost of cancer. For example, recent international studies have used administrative data to describe the excess cost of cancer in New Zealand [54], Canada [55], and England [56]. Our model, CancerCostMod does not seek to describe the excess cost of cancer compared to the general population, but rather, describe the distribution of costs for population groups experiencing poorer health outcomes. This paper aimed to 1) describe the development of our model, CancerCostMod; 2) to describe the total costs of cancer in Australia during the first 12-months post-diagnosis; and 3) to describe the distribution of the cost of cancer in Australia during the first 12-months post-diagnosis by population group.

We estimated that the total cost to the Australian healthcare system was $4.3 billion. Hospital episodes accounted the majority (77%) of the costs to the healthcare system. This is similar to the AIHW national report, which estimated that hospital admitted patient services accounted for 79% of the national healthcare system expenditure in 2008–09 [18]. Likewise, in other countries, admitted hospital services have been reported as contributing to the greatest proportion of cancer-related costs [55, 57, 58]. Initially, we described the total costs to the healthcare system, and the average costs per person to the healthcare system by cancer type, and population group. This initial analysis showed the average costs per person to the healthcare system were higher for each of our population groups of interest, compared to their reference group – Indigenous Australians, people living in remote areas, and people from the most disadvantaged quintile. We found that the total cost of cancer during the first 12-months post-diagnosis was significantly different for Indigenous Australians, people living in remote and very remote areas, and people living in areas of greater disadvantage. These differences in costs could be due to differences in health system use, which we will examine in future studies.

We estimated that the individual patient co-payment costs were $127 million in the first 12-months following diagnosis. The initial analysis found that the average co-payment costs were lower for Indigenous Australians, and people from the most disadvantaged quintile. After adjusting for age, sex, Indigenous status, rurality, IRSD quintiles, and broad cancer type, we found that the patient co-payment costs were lower for Indigenous Australians compared to non-Indigenous Australians, for people in inner and outer regional areas compared to metropolitan, and patient co-payment costs were greater for people from IRSD quintiles 4 and 5. These findings may be due to Australia’s universal healthcare system, and policies in place, such as the Medicare Safety Nets, the PBS Safety Net, and the CTG PBS Co-payment Programme to protect vulnerable population groups and people with higher healthcare co-payments. We will examine the differences in patient co-payments in more detail in future studies.

To our knowledge, this is the first study in Australia to use individual-level data to estimate the cost of cancer for Indigenous Australians. A previous report by AIHW and Cancer Australia reported that the average per person hospital expenditure for Indigenous Australians was half of that for non-Indigenous Australians [19]. However, this report used population-level data and only included hospitalizations for which cancer was the primary cause [19]. The advantage of using individual-level data allows us to determine if there are differences in health system use, and cost between Indigenous and non-Indigenous people. Further studies using CancerCostMod will examine these differences in more detail.

This is the first model in Australia, which aims to describe the healthcare system costs and patient co-payment costs for these population groups experiencing inequalities. Previous Australian studies have reported higher OOPE for rural patients with cancer [15, 16]. Recently, Newton et al. (2018) reported that the highest OOPE was due to surgery (23%), tests (20%), and accommodation (12%) [16]. Whereas, Gordon et al. (2009) reported travel costs accounting for the greatest proportion of patient OOPE (71%), followed by medical appointments (10%), and PBS co-payments (9%) [15]. Although CancerCostMod only estimates the patient co-payments for primary health care and prescription pharmaceuticals, it has the advantage of using administrative data, and thus is not subject to selection bias (recruitment and loss-to follow-up), or recall bias in recalling healthcare expenditure.

A strength of this study is that the base population includes everyone diagnosed with cancer in Queensland, and 3 years of follow-up data were obtained using linked administrative data. Our data have then been weighted to the Australian population to allow us to estimate the healthcare expenditure of cancer to the Australian healthcare system, and the patient co-payments for Australians diagnosed with cancer.

A common limitation of Australian health studies, is the completeness of Indigenous status on health records. We used multiple imputation to assign Indigenous status to missing records in our original QCR cohort. As reported, once weighted, our model included 2129 Indigenous Australians. There are currently no reliable data on the number of new cancer cases for Indigenous Australians for each jurisdiction in Australia. The most recent national AIHW data reports that in 2012, approximately 1343 new cancer cases were diagnosed in Indigenous Australians [59]. However, this estimate is based on five (of eight) jurisdictions only, in which the majority (90%) of Indigenous Australians live [59]. There is also varying levels of completeness of Indigenous status for each jurisdiction (from 2% unknown, to 18% unknown) [59]. Therefore, this national estimate may be underestimating the true incidence of cancer in Indigenous Australians [59]. One of the 2015 National Aboriginal and Torres Strait Islander Cancer Framework priorities is to “strengthen the capacity of cancer related services and systems to deliver good quality, integrates services that meet the needs of Aboriginal and Torres Strait Islander people” [60], and calls for improved recording of Indigenous status, and recommends the use of linked administrative data to look at patterns of care for Indigenous Australians in order to meet one of their priorities [60]. CancerCostMod uses individual-level data, which allows us to evaluate the service use and to quantify the cost of care for Indigenous Australians.

Administrative data have inherent weaknesses, primarily that the data are not collected for the purpose of research. For example, we were unable to estimate the cost of cancer by clinical staging, as this is not routinely collected by the QCR. The QCR does not collect individual or household financial information, therefore, we used aggregated area-level data to classify an individual’s level of socioeconomic disadvantage. We were also unable to analyze individual physiological, biological or clinical factors, which are considered by the treating specialist and may alter the suitability of different treatment options. The patient co-payment costs will be limited to costs incurred for MBS and PBS items, which excludes patient co-payment costs for private or non-prescription pharmaceuticals, private health insurance premiums, or hospital excess, or travel/accommodation costs. Finally, we sought to describe the cost of ED presentations for people with cancer. The IHPA first used the ED Classification System in the National Efficient Price Determination in 2012–13 [45]. The IHPA NHCDC annual reports include the average cost of each ED presentation from 2011, however, there have been several changes to the ED URG classification system and we have included the estimation of the costs of ED presentations separately to the hospital episodes.

## Conclusions

These findings are of interest to policy makers and healthcare providers, as they provide an evaluation of the total cost of cancer. To ensure an equitable healthcare system, it is first important to determine if there are any inequalities in relation to healthcare service use and expenditure amongst population groups whom experience poorer health outcomes. This paper describes the development of CancerCostMod, Australia’s first model of health service use, healthcare expenditure, and patient co-payment expenditure for people with cancer. CancerCostMod can be used to fill the gap by quantifying the current health system use, healthcare expenditure and patient co-payment costs for population groups experiencing poorer health outcomes - Indigenous people, people living in rural and remote areas, and socioeconomically disadvantaged persons. We found significant differences in healthcare expenditure and patient co-payments for the first 12-months following a cancer diagnosis for each of these population groups. CancerCostMod can be used to look at the cost from different perspectives for the first 3 years, with the potential to increase the cohort enrolment period and study period.

## Abbreviations

ABF: Activity based funding
ABS: Australian Bureau of Statistics
AIHW: Australian Institute of Health and Welfare
AR-DRG: Australian Refined Diagnostic-Related Group
ASGS: Australian Statistical Geography Standard
ED: Emergency department
EDIS: Emergency Department Information System
ICD-10-AM: International Classification of Diseases 10th Edition, Australian Modification
ICD-O: International Classification of Diseases for Oncology
IHPA: Independent Hospital Pricing Authority
IRSD: Index of Relative Socio-Economic Disadvantage
LGA: Local government area
MBS: Medicare Benefits Schedule
MI: Multiple imputation
NHCDC: National Hospital Cost Data Collection
NSW: New South Wales
OOPE: Out-of-pocket expenditure
PBS: Pharmaceutical Benefits Scheme
QCR: Queensland Cancer Registry
QHAPDC: Queensland Health Admitted Patient Data Collection
