# Correction to: CancerCostMod: a model of the healthcare expenditure, patient resource use, and patient co-payment costs for Australian cancer patients

**DOI:** 10.1186/s13561-019-0219-9

**Published:** 2019-01-19

**Authors:** Nicole Bates, Emily Callander, Daniel Lindsay, Kerrianne Watt

**Affiliations:** 1 College of Public Health, Medical and Veterinary Sciences (CPHMVS), James Cook University, Townsville, Australia; 2 Australian Institute of Tropical Health and Medicine (AITHM), James Cook University, Townsville, QLD Australia

**Correction to: Health Economics Review (2018) 8:28**

**https://doi.org/10.1186/s13561-018-0212-8**

## Correction

Following publication of the original article [1], the authors reported errors on their article.

In Tables 3 and 4 of this manuscript, the costs presented in the “hospital episodes”, “total cost to the health care system” and “average health care cost per person” columns were incorrect. Consequently, the numbers in the “Ratios” and “standard errors” columns related to the Hospital Episodes section of Table 5 were incorrect. The corrected Tables are shown below.

**Table 3:** Total cost of cancer during the first 12-months post-diagnosis by cancer type^1^

		COST TO THE AUSTRALIAN HEALTHCARE SYSTEM (AUD)						Patient co-payment^5^ (AUD)	
	N^2^	Hospital episodes^3^	ED presentations	MBS rebate^4^	PBS rebate	Total cost to the healthcare system	Average healthcare cost per person (SD)	Total	Average per person (SD)
All cancers combined	123,900	3,824,163,400	88,103,100	263,243,600	631,804,300	4,807,314,400	38,800 (42,900)	127,673,700	1000 (2000)
Prostate cancer	20,700	374,172,600	6,416,200	36,828, 600	38,650,600	456,068,000	22,000 (18,700)	16,324,000	800 (1200)
Breast cancer	15,400	377,396,200	6,744,700	36,690, 600	170,956,200	591,787,700	38,300 (31,300)	21,040, 900	1400 (1500)
Colorectal cancer	14,800	643,503,400	11,849,400	30,563,000	71,878,900	757,794,700	51,300 (39,800)	16,979,500	1100 (1800)
Blood and lymphatic system	13,300	616,132,000	13,286,600	40,503,100	176,947,100	846,868,700	63,600 (73,600)	24,655,400	1900 (4100)
Melanoma of the skin	12,300	70,844,800	1,575,700	23,174,800	12,506,700	108,102,000	8800 (13,400)	4,945,100	400 (500)
Tracheal, bronchus and lung cancer	11,100	434,675,600	15,618,400	24,164,600	45,331,000	519,789,700	46,800 (35,700)	10,168,700	900 (1700)
Digestive organs	10,100	433,725,600	12,668,100	20,303,100	31,119,600	497,816,400	49,400 (38,400)	11,273,000	1100 (2000)
Cancers of the urinary tract	6200	202,334,900	5,022,300	10,703,000	17,623,000	235,683,200	38,000 (32,600)	4,816,000	800 (1100)
Gynaecological cancers	5200	159,616,700	3,121,600	10,475,000	16,774,700	189,988,000	36,400 (32,700)	5,171,800	1000 (1600)
Head and neck	4000	141,306,700	2,478,200	10,264,700	9,019,700	163,069,400	40,500 (47,200)	2,374,400	600 (1200)
Other or ill-defined cancers	2700	76,240,900	2,316,900	4,295,300	6,051,400	88,904,500	32,800 (31,500)	2,239,700	800 (1700)
Thyroid and other endocrine glands	2600	55,221,900	799,400	4,195,500	3,631,300	63,848,200	24,600 (35,800)	1,180,600	500 (600)
Eye, brain and CNS	1900	114,903,700	3,113,900	3,324,700	17,342,800	138,685,000	71,800 (73,200)	2,830,000	1500 (2300)
Mesothelioma, Kaposi Sarcoma, and soft tissue	1600	69,741,200	1,882,200	3,245,200	9,187,200	84,055,900	51,500 (47,800)	2,080,100	1300 (2200)
Male genital organs, exc prostate	900	19,635,700	678,900	2,190,200	2,267,400	24,772,300	26,800 (30,900)	643,200	700 (1200)
Other skin cancer	800	15,355,100	283,300	1,749,400	1,456,800	18,844,600	22,700 (30,200)	541,900	700 (1100)

^1^Weighted results reported, and rounded to the nearest 100

^2^Excluding broad cancer types with less than 500 individuals (‘bone’, and ‘other thoracic and respiratory organs’)

^3^Admitted and non-admitted hospital episodes

^4^MBS rebate, excluding items included as non-admitted hospital episodes

^5^MBS and PBS patient co-payment

**Table 4:** Total cost of cancer for the first 12-months post-diagnosis, by population groups^1^

		COST TO THE AUSTRALIAN HEALTHCARE SYSTEM (AUD)						Patient co-payment^4^ (AUD)	
	n	Hospital episodes^2^	ED presentations	MBS rebate^3^	PBS rebate	Total cost to the healthcare system	Average healthcare cost per person (SD)	Total	Average per person (SD)
Weighted total	123,900	3,824,163,400	88,103,100	263,243,600	631,804,300	4,807,314,400	38,800 (42,900)	127,673,700	1000 (2000)
Indigenous status									
Non-Indigenous Australian	121,800	3,749,654,000	85,643,100	259,009,200	623,618,600	4,717,924,900	38,700 (42,800)	126,913,100	1000 (2000)
Indigenous Australian	2100	74,509,400	2,459,900	4,234,500	8,185,700	89,389,500	42,700 (48,800)	760,600	400 (800)
Rurality^5^									
Metropolitan	58,500	1,738,821,800	38,505,200	125,884,200	306,909,000	2,210,120,300	37,800 (39,900)	65,570,600	1100 (1900)
Regional	54,500	1,721,146,800	42,165,900	116,837,100	270,755,900	2,150,905,700	39,500 (43,800)	52,061,400	1000 (1900)
Remote	10,200	352,165,600	6,805,500	19,718,200	52,844,900	431,534,200	42,200 (53,800)	9,831,600	1000 (2500)
IRSD^6^ Quintiles^5^									
Q1 (Most disadvantaged)	11,300	387,822,900	11,248,900	21,232,900	47,295,500	467,600,300	41,400 (44,100)	9,226,300	800 (1900)
Q2	5700	183,953,200	3,180,500	11,653,000	28,801,500	227,588,200	39,800 (43,700)	4,900,200	900 (1800)
Q3	19,900	635,646,500	14,727,500	41,726,900	102,484,100	794,585,000	39,900 (48,500)	17,757,000	1100 (1800)
Q4	56,000	1,715,299,900	42,010,300	128,352,200	288,661,500	2,174,323,900	38,800 (42,000)	59,401,200	1100 (1800)
Q5 (Least disadvantaged)	30,200	889,411,700	16,309,300	59,474,600	163,267,300	1,128,462,900	37,300 (40,200)	36,179,100	1200 (2000)

^1^Weighted results reported, and rounded to the nearest 100

^2^Admitted and non-admitted hospital episodes

^3^MBS rebate, excluding items included as non-admitted hospital episodes

^4^MBS and PBS patient co-payment

^5^Excluding individuals with missing postcodes

^6^Index of Relative Socio-Economic Disadvantage

**Table 5:** Five generalized linear models of the cost of cancer for the first 12-months^1^

	Hospital episodes^2^		ED presentations		MBS rebate^3^		PBS rebates		Patient co-payment^4^	
	Ratio	Estimate (SE)	Ratio	Estimate (SE)	Ratio	Estimate (SE)	Ratio	Estimate (SE)	Ratio	Estimate (SE)
**Intercept**		8.5519 (0.0483) ***	–	5.4363 (0.0728) ***	–	5.7499 (0.0382) ***	–	6.8536 (0.0721) ***	–	4.7398 (0.0593) ***
**Age**	1.01	0.0062 (0.0005) ***	1.01	0.0103 (0.0008) ***	1.00	−0.0021 (0.0004) ***	0.99	−0.0109 (0.0008) ***	1.00	0.0033 (0.0006) ***
**Female**	0.88	−0.1267 (0.0167) ***	0.95	−0.0537 (0.0262) *	0.95	−0.0551 (0.0129) ***	0.85	−0.1597 (0.0234) ***	0.98	**−0.0214 (0.0192)**
**Indigenous Australians**	1.02	0.0220 (0.0525)	1.23	0.2095 (0.0752) **	0.92	−0.0888 (0.0402) *	0.82	−0.1928 (0.0735) **	0.39	−0.9315 (0.0619) ***
**Inner and outer regional area**	1.04	0.0364 (0.0165) *	1.02	**0.0196 (0.0275)**	0.99	**−0.0138 (0.0126)**	1.04	**0.0366 (0.0231)**	0.94	−0.0571 (0.0189) **
**Remote and very remote area**	1.10	0.0947 (0.0268) ***	1.03	**0.0322 (0.0454)**	0.90	−0.1092 (0.0207) ***	1.10	0.0912 (0.0381) *	0.98	**−0.0219 (0.0311)**
**IRSD**^5^ Q2	1.02	0.0214 (0.0373)	1.00	**0.0004 (0.0626)**	1.07	0.0675 (0.0289) *	1.05	**0.0474 (0.0519)**	1.00	**−0.0026 (0.0432)**
**IRSD**^5^ Q3	0.98	−0.0232 (0.0279)	1.01	**0.0097 (0.0420)**	1.08	0.0805 (0.0216) ***	1.11	0.1053 (0.0389) **	1.00	**−0.0020 (0.0324)**
**IRSD**^5^ Q4	0.95	−0.0510 (0.0250) *	0.98	**−0.0215 (0.0368)**	1.18	0.1642 (0.0193) ***	1.17	0.1582 (0.0348) ***	1.23	0.2033 (0.0289) ***
**IRSD**^5^ Q5 (least disadvantaged)	0.93	−0.0700 (0.0293) *	0.94	**−0.0603 (0.0467)**	1.00	**0.0016 (0.0226)**	1.19	0.1703 (0.0411) ***	1.32	0.2746 (0.0338) ***

*p-value < 0.05

**p-value < 0.01

***p-value < 0.001

^1^Adjusted for age at diagnosis, sex, Indigenous status, rurality, IRSD quintiles, and broad cancer type

^2^Admitted and non-admitted hospital episodes

^3^MBS rebate, excluding items included as non-admitted hospital episodes

^4^MBS and PBS co-payment is the cost to the patient

^5^Index of Relative Socio-Economic Disadvantage

Subsequently, the following sentences needed to be corrected. Corrected content is shown in bold:

## Abstract, Results, Discussion

The total initial cost associated with newly diagnosed cancer for the healthcare system is **$4.8** billion. Hospital episodes accounted for **80%** of the healthcare expenditure, followed by PBS (**13%**) and MBS (**5%**).

## Results, subheading “Results of Cost of cancer during the first 12-months post-diagnosis by population group”, 2nd paragraph:

Indigenous Australians had significantly higher costs for ED presentations (23% higher), but significantly lower costs for MBS rebates (8% lower), PBS rebates (18% lower), and patient co-payments (61% lower) compared to non-Indigenous Australians. [**The words “for hospital episodes (22%)” have been removed from the sentence**].

The costs for hospital episodes were significantly higher in the first 12-months post-diagnosis with increasing remoteness, **4%** for people living in inner and outer regional areas, and **10%** higher for people living in remote and very remote areas compared to those living in metropolitan areas. [**these percents were previously reported as 6% and 15% higher, respectively**].

Compared to those living in the most disadvantaged areas (IRSD Q1), those in **quintiles 4–5** had decreasing costs associated with hospital episodes [**quintiles 3–4 were previously reported**].
